# China’s integration in the Asia-Pacific regional economic cooperation

**DOI:** 10.3389/fpsyg.2022.951413

**Published:** 2022-09-29

**Authors:** Junhua Wei, Yue Gao, Ehsan Elahi

**Affiliations:** School of Economics, Shandong University of Technology, Zibo, Shandong, China

**Keywords:** Asia-Pacific, regional economic integration, cooperation, trade, China

## Abstract

China is actively deepening integration into economic cooperation in the Asia-Pacific region. The Regional Comprehensive Economic Partnership Agreement (RCEP) has come into force and China is one of its members. Furthermore, China is applying to join the Comprehensive and Progressive Agreement for Trans-Pacific Partnership (CPTPP). This study uses the Global Trade Analysis Project (GTAP) model to measure the impact of the RCEP and CPTPP on Gross Domestic Production (GDP), import, export, terms of trade, and social welfare of major economies under various scenarios, as well as the competitive effects and complementarity of the RCEP and CPTPP. We found that the CPTPP with China’s accession and the RCEP will complement and strengthen each other and that the members of the two agreements can obtain substantial benefits. If China and the United States join the CPTPP, China’s import growth rate will be higher than its exports. This would transmit growth to other nations and help bridge the trade gap between China and the United States.

## Introduction

While the multilateral integration process of the World Trade Organization is facing serious difficulties, regional economic integration is getting a chance to boom. Asia-Pacific, for instance, has witnessed rapid integration of its regional economy. China has been actively promoting and strengthening economic cooperation with nations in the Asia-Pacific region. The Regional Comprehensive Economic Partnership Agreement (RCEP) has been signed and entered into force. In addition, China is actively applying to join the Comprehensive and Progressive Agreement for Trans-Pacific Partnership (CPTPP). Economic integration in the Asia-Pacific region will exert tremendous influence on China and other nations.

As the world’s largest free trade area (FTA), the RCEP provides development opportunities for its member states. With the joint efforts of the Association of Southeast Asian Nations (ASEAN), China, Japan, South Korea, and other nations, the RCEP was officially signed in November 2020 after several rounds of negotiation and consultation. The RCEP is the FTA with the largest population, the most diversified membership, and the largest economic scale in the world. Its purpose to broaden and deepen economic integration in the region, strengthen economic growth and equitable economic development, and advance economic cooperation, through this Agreement, which will build upon existing economic linkages among the Parties. It seeks to establish clear and mutually advantageous rules to facilitate trade and investment, including participation in regional and global supply chains. The agreement is intended to strengthen economic dependence among RCEP members, help establish a unified free market, improve the effective allocation of resources in the Asia-Pacific region, and bring development opportunities for China and other nations.

Likely to the RCEP, the CPTPP is an important regional integration agreement in the Asia-Pacific region. The predecessor of CPTPP was the Trans-Pacific Partnership Agreement (TPP), initiated by the United States. During the tenure of former United States President Trump, the United States announced its withdrawal from TPP. After the United States withdrew from the TPP, more than 20 provisions of the TPP advocated by the United States were canceled, as a shortened form of the TPP, the CPTPP was officially signed in March 2018. The CPTPP is the fourth-largest FTA in the world. It has 11 member nations, covers about 500 million people, and is a high-standard FTA, with wider coverage and stricter rules than the RCEP. It has had the highest level of economic integration and trade freedom globally. To a great extent, it will guide international economic and trade rules in the future.

In September 2021, China submitted a formal application for accession to the CPTPP. For the United States, it is also possible to rejoin the CPTPP. In this context, it is particularly important to study the potential impact of the return of the United States into the CPTPP and the simultaneous entry of China and the United States into the CPTPP. The Global Trade Analysis Project (GTAP) will be used to measure the potential impact of the RCEP and CPTPP, project the impact of China’s accession to the CPTPP, and analyze the expected impact of simultaneous accession of China and the United States to the CPTPP, as well as the competitive effects and complementarity of RCEP and CPTPP.

There are three main contributions of the study. Firstly, after China accedes to the CPTPP, China will act as a bridge between the RCEP and CPTPP, and these two FTAs will complement and strengthen each other. Second, if both China and the United States join the CPTPP, the huge internal market of the CPTPP will enable China and the United States to achieve win-win results, and the members of the two FTAs will obtain much greater benefits. Under some scenarios, the growth rate of China’s imports will be higher than its exports, stimulating growth in other nations. Thirdly, if both China and the United States join the CPTPP, this will significantly increase bilateral trade and help to achieve a trade balance between the two countries.

## Literature review

Studies of the RCEP have revealed positive effects for China. RCEP, as the largest FTA in the world, has received extensive attention from scholars. Some scholars have made qualitative analyses on the importance of RCEP for China from different research perspectives. Wang and Wang (2021) concluded that the RCEP is conducive to developing China’s regional value chain. Liu (2021) concluded that the RCEP has played an important role in both promoting China’s exports and imports and driving enterprises to enhance competitiveness. Shen and Li (2020) concluded that the RCEP would help China reform its economic system and rules, and further promote China’s market opening. Liu et al. (2021) concluded that tariff reductions among RCEP members would improve the social welfare of China, Japan, South Korea, and Australia, and enhance the overall benefits of social development.

Based on the GTAP model and China’s regional computable general equilibrium model, Zhang and Zhou (2021) found that RCEP positively impacted China’s social welfare, imports and exports. However, the impact was different for different reasons, regions and industries. Lv and Li (2018), Zhang and Zhan (2018), and Li et al. (2020) simulated the economic impact of the RCEP by using the GTAP model and concluded that the RCEP would have a positive impact on China’s economy. Based on the GTAP model, Lu et al. (2021) analyzed the economic effects of the RCEP and found that it could promote a high-quality open economy. All of the above studies used the GTAP model as the main analytical tool, and others used other tools. For example, Kohl (2014) and Rajan and Huang (2016) used the gravity model and found that the RCEP would increase China’s growth and trade.

The impact of the CPTPP on the Asia-Pacific region and how China should respond to it has become the focus of discussion (Petri and Plummer, 2012; Solís and Katada, 2015). Some papers concluded that accession to the CPTPP would provide China with benefits and challenges. On the one hand, if China joins the CPTPP, GDP, manufacturing employment, and social welfare will increase on a large scale (Li et al., 2021). The high-standard rules of the CPTPP will set an example for China to follow (Zhang, 2021); and the agreement will be conducive to China’s expansion of trade and high-quality growth (Feng, 2021). On the other hand, the CPTPP involves issues such as “non-market economy status,” “competition neutrality policy,” “industrial subsidy policy,” and “market/institutional barriers and constraints,” which are the focus of the conflicts between China and developed nations (Chen, 2021). Furthermore, the United States and Japan may block China’s accession to the CPTPP (Zhang and Wang, 2021).

Some studies (e.g., Yu and Jiang, 2021) have compared the provisions of the RCEP and CPTPP and analyzed their differences. For example, Yu et al. (2021) found a large gap between the two agreements in terms of trade in goods, services, investment, and dispute settlement. Quan (2021) compared and analyzed the differences in the rules on trade in services, especially the differences in regulations on financial services and telecommunications services, as well as the advantages and disadvantages of accession to the CPTPP for China’s service industry.

While previous studies have mainly focused on the impact of the RCEP and CPTPP and the comparison of their provisions (e.g., Lv and Li, 2018; Guan and Liang, 2019), research is lacking on the different impacts of consecutive and simultaneous accession of China and the United States to CPTPP, as well as on the complementarity and competitive ways in which the RCEP and CPTPP affect each other. This paper’s contribution is to study the impact of the RCEP and CPTPP on China and other nations under various scenarios.

## Materials and methods

The GTAP model is a multi-country and multi-sector general equilibrium model designed based on neoclassical economic theory. GTAP has been widely used for trade policy analysis. The GTAP model comprises the main operation program (RunGTAP) and the database software (GTAPAgg). The GTAPAgg database contains many kinds of real economic data such as imports and exports, economic growth, and industrial output in many nations or regions of the world. This paper uses the 10th edition database of the GTAP model, and its base year is 2014. In this paper, the research on the impact of the RCEP and CPTPP will be carried out using the GTAP model.

### Sector classification in the global trade analysis project model

The 10th edition of the GTAPAgg database contains data on 65 industries, mainly derived from the International Standard Industrial Classification (ISIC) and the Cooperative Patent Classification (CPC). This paper divides these industries into 10 categories: grains and crops, animal husbandry and meat products, natural resources, processed food, textile and garment industry, light industry, heavy industry, public utilities and construction, transportation and communication industry, and other service industries. Of the 10 categories, grains and crops, animal husbandry and meat products, natural resources, and processed food belong to the primary industry. Textile, garment, light, and heavy industries belong to the manufacturing industry. Similarly, the public utilities and construction, transportation and communication industry, and other services belong to the tertiary industry (Table 1).

**TABLE 1:** Classification of industries.

Categories	Industries
Grains and crops	Paddy rice, wheat, cereal grains nec, vegetables, fruit, nuts, oil seeds, sugar cane, sugar beet, crops nec, plant-based fibers, and processed rice
Animal husbandry and meat products	Bovine cattle, sheep and goats, horses, animal products nec, raw milk, wool, silk-worm cocoons, Bovine meat products, and meat products nec
Natural resources	Forestry, fishing, coal, oil, gas, and minerals nec
Processed food	Vegetable oils and fats, dairy products, sugar, food products nec, beverages, and tobacco products
Textile and garment industry	Textiles and wearing apparel
Light industry	Leather products, wood products, paper products, publishing, metal products, motor vehicles and parts, transport equipment nec, and manufactures nec
Heavy industry	Petroleum, coal products, chemicals, rubber, plastic products, mineral products nec, ferrous metals, metals nec, electronic equipment, machinery, and equipment nec
Public utilities and construction	Electricity, gas manufacture, distribution, water, and construction
Transportation and communication industry	Trade, transport nec, water transport, air transport, and communication
Other services	Financial services nec, insurance, business services nec, recreational and other services, public administration, defense, education, and health

Industries classification is from the 10th edition of the GTAPAgg database. This paper clusters these industries into 10 categories; “nec” means “not elsewhere classified”.

### Regions setting in the global trade analysis project model

For convenience, this paper divides the economies into different groups. Currently, the RCEP comprises 15 countries, including 10 ASEAN countries (Malaysia, Indonesia, Thailand, Philippines, Singapore, Brunei, Vietnam, Laos, Myanmar, and Cambodia), China, Japan, South Korea, New Zealand, and Australia. The CPTPP now has 11 members particularly Japan, Canada, Australia, Chile, New Zealand, Singapore, Brunei, Malaysia, Vietnam, Mexico, and Peru. This paper lists separately the economies or organizations with the greatest influence on the world economy, including China, United States, Japan, South Korea, and the European Union, each of which acts as a separate group. The nations that belong to the RCEP and CPTPP are grouped: Australia, Malaysia, Singapore, Brunei, Vietnam, and New Zealand. The nations that are members of the RCEP but not the CPTPP are placed into one group: Indonesia, Philippines, Thailand, Cambodia, Laos, and Myanmar. The nations that are members of the CPTPP but not the RCEP are placed into one group: Canada, Chile, Mexico, and Peru. At last, the remaining nations in GTAPAgg are classified into one group (Table 2).

**TABLE 2:** Regions settings.

Abbreviation	Region name	Nations included
CHN	China	China
USA	United States	United States
JPN	Japan	Japan
KR	Korea	Korea
RCCP	Members of both RCEP and CPTPP	Australia, Malaysia, Singapore, Brunei, Vietnam, and New Zealand
OTRC	RCEP members but not CPTPP members	Indonesia, Philippines, Thailand, Cambodia, Laos, and Myanmar
OTCP	CPTPP members but not RCEP members	Canada, Chile, Mexico, and Peru
EU	European Union	The EU now has 27 countries
Rest	Other regions or nations in the world	Remaining countries in GTAPAgg

### Scenario setting

This paper uses GTAP to simulate the impact of reducing tariffs and technical barriers in the RCEP and CPTPP agreements. Five scenarios exist particularly, scenario 1; tariffs of the RCEP are reduced to 0 and the technical barriers of the RCEP are reduced by 10%. Scenario 1 is mainly set to simulate the changes affecting nations in the Asia-Pacific region and other regions after the RCEP enters into effect. Scenario 2; tariffs of both the RCEP and CPTPP are reduced to 0 and their technical barriers are reduced by 10% among their respective members. Scenario 2 is set to study the impact of the CPTPP, especially the impact of the CPTPP on RCEP members to investigate the competitive and complementarity relationship between the CPTPP and RCEP. Scenario 3; using the setting of scenario 1, assume that China joins the CPTPP, and CPTPP tariffs are reduced to 0, and its technical barriers are reduced by 10%. Scenario 3 is used to study the impact of China’s accession to CPTPP, and to analyze the impact of China’s simultaneous accession to the CPTPP and RCEP. Scenario 4; using the setting of scenario 1, assume that the United States joins the CPTPP, the CPTPP tariffs are reduced to 0, and the technical barriers are reduced by 10%. Scenario 5; using the setting of scenario 1, assume that both China and the United States join the CPTPP, the tariffs of CPTPP are reduced to 0, and its technical barriers are reduced by 10%. This scenario is set to simulate the impact of the simultaneous entry of China and the United States into the CPTPP (Table 3).

**TABLE 3:** Scenario settings.

Scenario	Scenario settings
Scenario 1	Tariffs of RCEP are reduced to 0 and technical barriers of RCEP are reduced by 10%
Scenario 2	Tariffs of both RCEP and CPTPP are reduced to 0 and technical barriers are reduced by 10%
Scenario 3	With the setting of Scenario 1, assume China joins CPTPP, tariffs of CPTPP are reduced to 0, and technical barriers of CPTPP are reduced by 10%
Scenario 4	With the setting of Scenario 1, assume the United States joins CPTPP, tariffs of CPTPP are reduced to 0, and technical barriers of CPTPP are reduced by 10%
Scenario 5	With the setting of Scenario 1, assume that both China and the United States join CPTPP, tariffs of CPTPP are reduced to 0, and technical barriers of CPTPP are reduced by 10%

## Results and discussion

In this study, we simulated scenario 1 to scenario 5 through the 10th version of the GTAP model, mainly focusing on the changes in GDP, the amount of import and export, terms of trade, social welfare of each economy, and the import and export of each sector of China’s manufacturing industry.

### Impact on gross domestic production

The impact on the GDP of each economy under each scenario is given in Table 4. Before the signing of the RCEP, some members had formed FTAs, but China, Japan, and South Korea had not reached an FTA among them. The RCEP is equivalent to creating free trade relations among China, Japan, and South Korea, which is of historical significance.

**TABLE 4:** Impact on gross domestic production (GDP) (%).

Area	Scenario 1	Scenario 2	Scenario 3	Scenario 4	Scenario 5
CHN	2.12	–0.36	2.91	–2.36	3.19
USA	–1.75	–0.39	–2.7	2.25	0.89
JPN	7.38	2.51	7.75	2.98	7.41
KR	9.69	–0.31	9.28	–2.09	6.71
RCCP	5.18	1.8	6.92	2.29	4.99
OTRC	3.81	–0.46	3.44	–2.16	1.52
OTCP	–1.52	0.76	1.1	8.32	6.71
EU	–1.33	–0.16	–1.51	–1.67	–3.68
Rest	–1.79	–0.24	–1.98	–1.78	–4.2

From the simulation results of Scenario 1, it can be seen that the RCEP leads to growth in the GDP of member nations, including nations that are members of both the RCEP and CPTPP and nations that are members of RCEP only. While the growth rates are 9.69% in South Korea and 7.38% in Japan, the increase in China’s GDP is only 2.12%. The GDP of economies other than RCEP members is negatively affected. This includes the United States, and its GDP decreased by 1.75%, followed by nations that are CPTPP members only, and their GDP growth dropped by 1.52%. The GDP of the EU and other regions or nations is also negatively affected. It can be seen that the RCEP can promote the GDP of its members to varying degrees by reducing tariffs and technical barriers but brings about varying degrees of negative impact on non-members.

According to the simulation results of Scenario 2, CPTPP can promote GDP growth among member nations, especially in Japan, where GDP growth increases by 2.51%, while most non-members of CPTPP suffer losses, including China, the United States, South Korea, and nations that are RCEP members only. By comparing the simulation results of Scenario 2 and Scenario 1, it can be found that the GDP growth rate in Scenario 2 is lower than that in Scenario 1 for China, Japan, and South Korea, which includes nations that are members of both the RCEP and CPTPP and nations that are members of RCEP only. Therefore, the CPTPP offsets part of the benefits brought about by the RCEP, and there exists competition between the CPTPP and RCEP, which further confirms that regional economic integration has some exclusivity features.

The simulation results of Scenario 3 show that China’s accession to CPTPP can increase China’s GDP growth by 2.91%. Both CPTPP and RCEP members will benefit from China’s accession as well. For example, the GDP growth of South Korea and nations that are RCEP members will increase by 9.28 and 3.44%, respectively. Nations that are both RCEP and CPTPP members will achieve an increase of 6.92%, especially Japan, whose GDP growth will increase by 7.75%. The United States and EU, which are neither RCEP nor CPTPP members, will suffer losses, with GDP growth falling by 2.7 and 1.51%, respectively. Therefore, from the comparison between Scenario 3 and Scenario 2, we can see that CPTPP with China’s accession and RCEP complement each other and that the members of the two agreements can obtain benefits. As the world’s second-largest economy and the largest nation in the trade of goods, China is already maintaining close economic and trade ties with most members of the RCEP and CPTPP. When China joins RCEP and CPTPP, it will be able to produce a bridging effect among RCEP and CPTPP members.

Scenario 4 is the case where the United States joins the CPTPP, but China does not. Compared with Scenario 2, the simulation results of Scenario 4 show that accession of the United States to the CPTPP will produce benefits for both parties, with overall GDP growth for CPTPP members and 2.25% GDP growth for the United States. For non-CPTPP members, United States accession to CPTPP will produce a negative impact. For example, the GDP of China, South Korea, and other nations (RCEP members) reduced by more than 2%. Therefore, the CPTPP joined by the United States has an inhibitory effect on non-CPTPP members.

Scenario 5 examines the situation in which China and the United States join the CPTPP. A comparison of the first two lines in Scenarios 3, 4, and 5 depict that when China joins the CPTPP but the United States does not, the GDP growth of China will increase, while the GDP of the United States will decrease. When the United States joins the CPTPP but China does not, the result is just the opposite: an increase in the United States GDP growth and a decrease in China’s GDP growth. When China and the United States joined the CPTPP, the two nations achieved a win-win result, positively affecting their GDP growth. The simulation results of scenario 5 show that the GDP growth of Japan and South Korea is significant, reaching 7.41 and 6.71%, respectively. The GDP growth of nations that are CPTPP members only, RCEP and CPTPP members, and RCEP members only will increase by 6.71, 4.99, and 1.52%, respectively. Therefore, China and United States, as the world’s top two economies, will promote the integration process of the Asia-Pacific region if they join CPTPP. This would be conducive to the optimal allocation of resources in the region.

### Impact on imports and exports

Table 5 lists the changes in exports and imports of each economy under each scenario. The simulation results of scenario 1 show that after the RCEP reduces tariffs and technical barriers, the exports and imports of all members will increase. While the exports and imports of non-members will decrease. This demonstrates the presence of trade creation and trade diversion effects. The simulation results of scenario 2 show that CPTPP has trade creation and diversion effects.

**TABLE 5:** Impact on imports and exports (%).

Area		Scenario 1	Scenario 2	Scenario 3	Scenario 4	Scenario 5
CHN	Import	9.9	–0.76	11.95	–3.92	15.36
	Export	5.49	–0.22	6.52	–0.71	9.8
USA	Import	–3.55	–0.88	–5.71	8.45	9.13
	Export	–0.31	–0.18	–0.9	5.59	8.2
JPN	Import	14.54	3.76	15.28	5.65	15.85
	Export	5.92	0.54	6.26	2.38	8.12
KR	Import	14.84	–0.41	14.58	–2.62	11.54
	Export	8.46	–0.18	8.36	–1.17	7.08
RCCP	Import	7.18	2.42	9.91	3.82	8.07
	Export	4.44	1.56	6.32	2.68	5.45
OTRC	Import	9.15	–0.68	8.78	–2.86	6.06
	Export	6.36	–0.42	6.03	–1.46	5.13
OTCP	Import	–1.73	1.1	2.39	13.17	11.75
	Export	–0.48	0.35	0.62	6.05	5.44
EU	Import	–1.7	–0.21	–1.9	–2.07	–4.42
	Export	–0.84	–0.1	–0.91	–0.87	–1.87
Rest	Import	–2.88	–0.35	–3.18	–2.57	–6.34
	Export	–1.66	–0.22	–1.8	–1.18	–3.09

The simulation results of scenario 3 shows that after China acceded to the CPTPP, the exports and imports of RCEP and CPTPP members will increase significantly, indicating an augmented trade creation effect and an inhibited trade diversion effect in the RCEP and CPTPP. Therefore, after China’s accession, the complementarity between CPTPP and RCEP was strengthened, and their competitiveness was weakened. If China is a member of the RCEP and CPTPP, it can act as a bridge to promote trade among the members of both FTAs.

In the next step, we compared the simulation results of scenario 4 with scenario 2. When the United States joins the CPTPP and China does not, it will promote the exports and imports of the United States and CPTPP members and inhibit the exports and imports of RCEP members. Therefore, when the United States joins the CPTPP, but China does not, it strengthens the trade diversion effect of CPTPP and turns the members’ trade more inward.

Scenario 5 examines the situation when China and United States join the CPTPP simultaneously. Results reveal expansion in exports and imports for China, United States, and RCEP and CPTPP members. Compared with the situation in which neither China nor the United States joins the CPTPP, the imports and exports of the RCEP and CPTPP members will increase when China and the United States join CPTPP simultaneously. A comparison between scenarios 3, 4, and 5 shows that the entry of China and the United States into the CPTPP significantly promotes the exports and imports of the two nations. While the entry of just one nation into the CPTPP promotes the exports and imports of this nation but reduces that of the other nation. Therefore, the simultaneous entry of China and the United States will produce a win-win result and will play an important role in promoting trade between RCEP and CPTPP members.

In scenarios 1, 3, and 5, the growth of China’s imports is much higher than that of exports. Therefore, these circumstances are conducive to reducing China’s trade surplus and promoting trade balance. In this way, China transmits growth to other nations.

### Impact on trade terms

Table 6 shows the changes in economies’ terms of trade under each scenario. Terms of trade measure the number of goods of other economies that can be exchanged for one unit of goods exported by an economy. The higher the terms of trade number, the more benefits an economy can gain from foreign trade. The simulation results of scenario 1 show that the RCEP improves member nations’ trade terms. Japan’s terms of trade number improves by 4.55% followed by South Korea with an improvement of 3.75%. The improvement in China’s terms of trade is 0.34%, which is lower than that of other member nations in the RCEP. The RCEP has a negative effect on the terms of trade of non-members and has the greatest negative impact on the United States. Nations that are CPTPP members only, the European Union, and the other nations are negatively affected to varying degrees.

**TABLE 6:** Impact on trade terms (%).

Area	Scenario 1	Scenario 2	Scenario 3	Scenario 4	Scenario 5
CHN	0.34	–0.33	0.83	–1.17	1.74
USA	–1.14	–0.36	–2.12	1.33	0.43
JPN	4.55	1.49	4.72	2.41	5.09
KR	3.75	–0.19	3.5	–0.71	2.62
RCCP	2.21	0.97	3.01	1.36	2.16
OTRC	0.86	–0.4	0.29	–0.92	0.01
OTCP	–0.62	0.45	0.71	3.94	2.96
EU	–0.25	–0.02	–0.31	–0.28	–0.73
Rest	–1.03	–0.15	–1.15	–0.72	–2

The simulation results of Scenario 2 show that the CPTPP can improve the terms of trade of member nations, especially Japan, whose terms of trade will increase by 1.49%. While the terms of trade of non-members of CPTPP (including China, United States, and South Korea) will deteriorate. Comparing scenario 1 with scenario 2, it can be seen that the improvement of RCEP members in scenario 2 is lower than that in scenario 1. Therefore, CPTPP has an adverse impact on the trade terms of RCEP members.

The simulation results of scenario 3 found that if China joins the CPTPP, the terms of trade will be greatly improved, and the members of the RCEP and CPTPP will also be positively affected. For example, the trade terms of South Korea and nations that are RCEP members only will be improved by 3.5 and 0.29%, respectively, while the improvement for CPTPP nations that are not RCEP members is 0.71%. The improvement for nations that are both RCEP and CPTPP members will reach 3.01%, of which Japan has the highest improvement, reaching 4.72%. The terms of trade of the United States, the European Union, and the other nations will be adversely affected. By comparing Scenario 3 with Scenario 2, it can be seen that if China joins CPTPP, the improvement of the terms of trade of RCEP and CPTPP members is higher than that in Scenario 2.

Scenario 4 simulates the situation in which the United States joins CPTPP but China does not. Comparing the simulated results in scenario 4 with those of scenario 2, it can be seen that after the United States joins CPTPP, there will be a greater degree of improvement in trade terms for its members. For non-CPTPP nations, the entry of the United States has a greater negative effect on trade terms, among which China is affected the most, with a negative effect of 1.17%.

Scenario 5 examines the situation of both China and the United States joining the CPTPP. When both China and United States join the CPTPP, the terms of trade between these two nations will improve. Comparing Scenario 5 with Scenarios 3 and 4, it can be seen that when China joins the CPTPP and the United States does not, China’s terms of trade will improve, but there is a deterioration in terms of trade for the United States. When the United States joins the CPTPP and China does not the terms of trade of the United States improve significantly, while China’s terms of trade are negatively affected. The simulation results of scenario 5 show that the trade terms of Japan and South Korea improve significantly, by 5.09 and 2.62%, respectively. The trade terms improve to varying degrees for nations that are members of RCEP and CPTPP, those that are RCEP members only, and those that are CPTPP members only.

### Impact on social welfare

In the GTAP model, the changes in social welfare are measured in equivalent variation. Table 7 simulates the changes in social welfare in Asia-Pacific economies under different scenarios. The simulation results of scenario 1 show that the RCEP improves the social welfare of its members. For example, China, Japan, and South Korea get an increase of United States $144.516, $96.389, and $62.416 billion, respectively. However, the RCEP has a negative impact on the social welfare of non-member nations.

**TABLE 7:** Impact on social welfare (United States $ billion).

Areas	Scenario 1	Scenario 2	Scenario 3	Scenario 4	Scenario 5
CHN	144.516	–10.648	174.293	–47.668	231.296
USA	–30.971	–9.719	–58.078	129.436	160.703
JPN	96.389	33.364	103.076	54.664	119.038
KR	62.416	–1.438	60.472	–6.144	52.941
RCCP	78.969	23.93	95.214	39.614	91.532
OTRC	40.731	–2.776	41.327	–7.053	34.58
OTCP	–8.665	12.912	39.03	115.526	125.264
EU	–22.578	–1.951	–26.919	–28.657	–68.46
Rest	–76.909	–13.67	–84.872	–56.265	–150.39

The simulation results of scenario 2 show that the CPTPP can improve the social welfare of its member nations. Japan has the largest growth, followed by both RCEP and CPTPP members, and then nations that are CPTPP members only, with inner cases of United States $33.364 billion, $23.930 billion, and $12.912 billion, respectively. However, comparing scenario 1 with scenario 2, it can be seen that the CPTPP reduces the social welfare of RCEP members.

Scenario 3 simulates the effect of China’s accession to the CPTPP. If China joins the CPTPP, its social welfare will be improved, and the RCEP and CPTPP member nations will also be positively affected. For example, the social welfare of South Korea and nations that are only RCEP members will increase by $60.472 and $41.327 billion, respectively. The social welfare of nations that are CPTPP members only and nations that are both RCEP and CPTPP members will increase by $39.030 and $95.214 billion, respectively. The social welfare of United States, European Union, and other nations will decrease by varying degrees. By comparing scenario 3 with scenario 2, it can be seen that the growth of social welfare of RCEP and CPTPP members after China’s accession to the CPTPP is higher than that in scenario 2, where China doesn’t join the CPTPP.

The simulation results of Scenario 4 show that when the United States joins the CPTPP but China does not, the social welfare of the United States will increase by $129.436 billion. The social welfare of Japan, the nations that are members of both RCEP and CPTPP, and those that are only CPTPP members will increase. But the social welfare of non-CPTPP members will be negatively affected, with China having the greatest adverse impact of a $47.668 billion loss. While South Korea, and nations that are only RCEP members will face a reduction of $6144 and $7053 million, respectively. Therefore, by comparing scenario 4 with scenario 2, it can be seen that United States accession to the CPTPP would have a negative impact on the social welfare of non-CPTPP members.

Scenario 5 analyzes the social welfare of Asia-Pacific economies when both China and the United States join CPTPP simultaneously. When both China and United States join CPTPP, the social welfare of both RCEP and CPTPP members will increase. Comparing scenario 5 with scenarios 3 and 4, it can be seen that the social welfare of China and the United States in scenario 5 will be greater than that in scenarios 3 and 4. Therefore, China and the United States can achieve win-win results if they join the CPTPP.

### Impact on the import and export of China’s manufacturing industries

**FIGURE 1:**
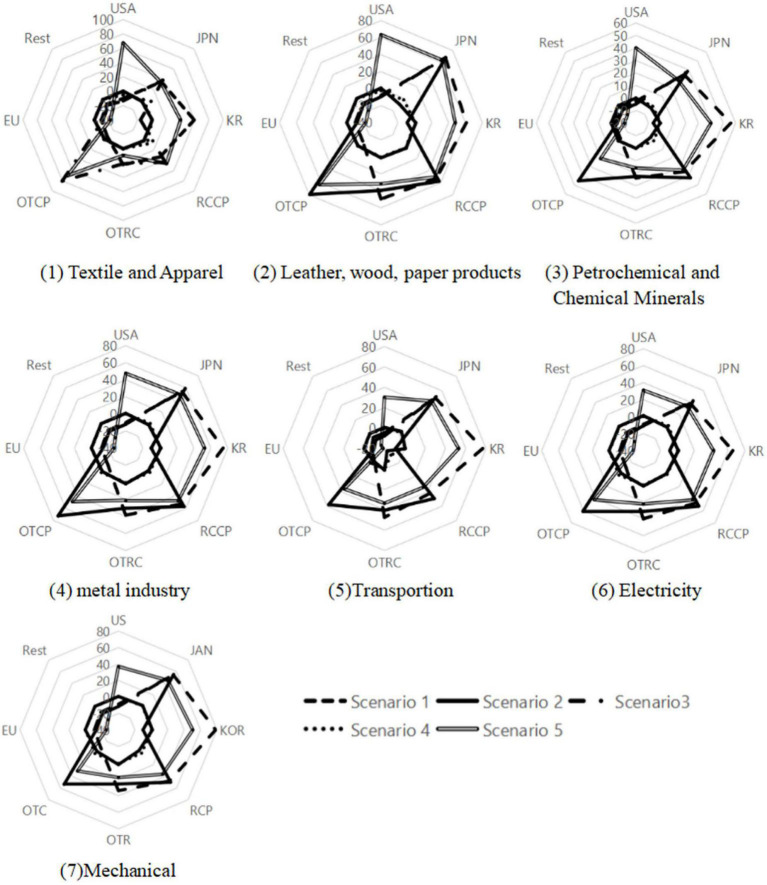
Impact on exports of China’s manufacturing industries (Manufacturing industries classification is from the 10th edition of the GTAPAgg database).

Figure 1 illustrates the impact on the exports of some of China’s manufacturing industries under different scenarios. We mainly focus on the simulation results of Scenario 1, Scenario 3, and Scenario 5. It can be seen from the simulation results of scenario 1 that after the RCEP takes effect, China’s exports to RCEP members increase significantly. The RCEP leads to the highest growth rate of China’s exports to South Korea followed by Japan, then nations that are members of RCEP and CPTPP. China’s exports to non-RCEP members decrease significantly, especially to United States and EU.

The simulation results of scenario 3 show a significant increase in China’s exports to CPTPP members, especially Japan, CPTPP members only, and members of both the RCEP and CPTPP. However, there’s a drastic decline in China’s exports to non-CPTPP member countries, particularly the United States, South Korea, and nations that are RCEP members only. Regarding industry composition, the export promotion impact on the leather, wood, paper products, textile and apparel industries is relatively strong, and that on petrochemical and chemical minerals is relatively weak.

Scenario 5 describes the effect of the accession of China and the United States to CPTPP. The simulation results show that exports to the Asia-Pacific region significantly increase in all sectors, especially in the electricity, transportation, mechanical, and metal industries. At the national level, when both China and United States join CPTPP, China’s exports to United States and CPTPP nations that are not RCEP members increased on a large scale.

**FIGURE 2:**
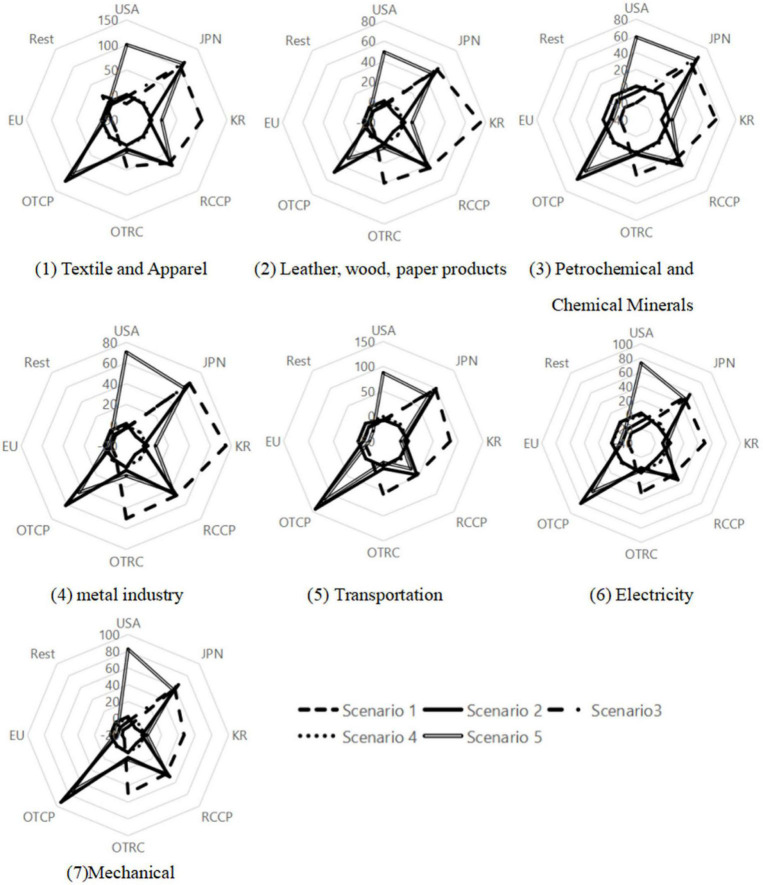
Impact on imports of China’s manufacturing industries (Manufacturing industries classification is from the 10th edition of the GTAPAgg database).

Figure 2 illustrates the impact of China’s accession to the CPTPP on different industries. The simulation results of scenario 1 show that China’s imports from RCEP members will increase significantly, especially in the metals and transportation industries; however, imports from non-RCEP members will decline. The simulation results of Scenario 3 show that if China joins the CPTPP, China’s imports from CPTPP members will increase significantly, while imports from non-CPTPP members will decline. According to the simulation results of scenario 5, after both China and the United States join CPTPP, China’s imports from both RCEP and CPTPP members will increase substantially. By comparing the simulation results of Scenario 5 in Figures 1, 2, it is found that the accession of China and the United States to the CPTPP results have a notable increase in bilateral trade with China importing more from the United States than it exports to the United States. Therefore, it is conducive to reducing China’s trade surplus with the United States.

## Conclusion and policy implications

### Main findings

In the current study, we used the GTAP model to measure the impact of the RCEP and CPTPP on GDP, imports, export, terms of trade, and social welfare of China and other economies. In addition, we also analyzed the impact of the simultaneous accession of China and the United States on the CPTPP. The findings of the study can be summarized as:

Firstly, the RCEP benefits its member nations by lowering tariffs and technical barriers, but non-member nations suffer some losses from the RCEP. When both the CPTPP and RCEP enter into force, some of the benefits induced by the RCEP will be offset. Therefore, there is a certain degree of competitiveness between the RCEP and CPTPP.

Secondly, if China becomes a member of both RCEP and CPTPP, as the world’s second-largest economy, China will act as a link between the members of the two FTAs. After China’s accession, the CPTPP and RCEP will complement and strengthen each other.

Thirdly, China and United States are respectively the world’s second and first largest economies; if both join the CPTPP, it will become the world’s largest FTA, and this huge regional internal market will bring economy of scale. The larger the FTA market, the greater the trade creation effect and the smaller the trade diversion effect. Therefore, nearly all members of the two FTAs can obtain substantial benefits. Furthermore, China and the United States can achieve win-win results.

In contrast with other literature in the same field, this paper finds some new ideas. In particular, the CPTPP with China’s accession and the RCEP will complement and strengthen each other. If both China and United States join CPTPP, China will transmit growth to other nations and help to bridge the trade gap between China and United States.

### Policy implications

Comprehensive and progressive agreement for trans-pacific partnership is an influential FTA in the Asia-Pacific region. China should pick up the pace to join the CPTPP. According to the research results of this study, if China joins the CPTPP, it will greatly improve China’s GDP, import and export growth, trade terms, and social welfare. With China’s accession, the CPTPP and RCEP will complement and reinforce each other, jointly enhancing the interests of the members of the two agreements. Therefore, China’s accession to the CPTPP would be beneficial to its interests and good for the interests of CPTPP and RCEP members.

These agreements can potentially strengthen cooperation between China and United States, and promote economic integration in the Asia-Pacific Region. If both China and United States join the CPTPP, China will become a member of both RCEP and CPTPP, and United States will be a member of both CPTPP and the United States-Mexico-Canada Agreement (referred as USMCA). China will act as a bridge between RCEP and CPTPP. Similarly, United States will act as a bridge between the CPTPP and USMCA. Therefore, a larger market with close economic and trade links will be formed through the two bridges. Consequently, China and United States will achieve a win-win result and bring greater development opportunities to other nations in the Asia-Pacific region.

China should undertake further institutional opening-up to promote reform. High-standard FTAs such as the CPTPP, USMCA, and Japan-EU Economic Partnership Agreement (EPA) have two characteristics in common. One is that the topics covered are very broad, and the standards of rules and institutions are strict; the other is that the provisions of these FTAs discriminate against non-members, effectively avoiding competition from nations outside the FTA. China has not yet participated in these high-standard FTAs and may face the risk of being marginalized if it does not promote domestic reforms, open up markets, counter the threats of “chain-breaking” and “decoupling,” and stabilize its position in the global value chain.

The study also has the following limitations. Firstly, this paper assumes that there is a possibility of China’s and the United States’ joining the CPTPP; in fact, China has applied for CPTPP membership. But there is also a possibility of their failure to join CPTPP. For example, the United States is engaged in the Indo-Pacific Economic Framework for Prosperity (IPEF), which might make the United States deviate from the CPTPP.

However, some conclusions still hold in similar scenarios even if China and/or the United States fail to join the CPTPP. For example, if one big nation becomes a member of two FTAs, it will act as a bridge, and these two FTAs will complement and strengthen each other; if two big nations join the same FTA, the huge internal market of the FTA will enable the two nations to achieve win-win results and cause the members of the FTA to obtain more benefits than before. Secondly, similar to many CGE studies, we assume a perfect market, where consumers pursue utility maximization and producers pursue profit maximization. This may deviate from reality by overlooking other factors determining the behavior of consumers and producers. This analysis would be improved if a dynamic version of the GTAP model were adopted instead of the static version. Lastly, the FTAs’ impacts in this study are modeled as a reduction in trade barriers, which captures only part of the FTAs’ impacts.

## Data availability statement

The raw data supporting the conclusions of this article will be made available by the authors, without undue reservation.

## Author contributions

JW and YG were responsible for data collection, arrangement of relevant literature, and data analysis. EE commented on the choice of the research topic and helped to write an original and final draft of the manuscript. All authors contributed to the article and approved the submitted version.
